# Neurological, Neurodevelopmental and Treatment Outcomes in Patients With Pyruvate Dehydrogenase Complex Deficiency

**DOI:** 10.1002/acn3.70510

**Published:** 2026-08-06

**Authors:** Antri Savvidou, Colin Reilly, Kalliopi Sofou, Sofia Ygberg, Erik A. Eklund, Karin Naess, Niklas Darin

**Affiliations:** ^1^ Department of Pediatrics, Institute of Clinical Sciences, Sahlgrenska Academy University of Gothenburg Gothenburg Sweden; ^2^ Department of Pediatric Neurology and Psychiatry, Sahlgrenska University Hospital Gothenburg, Region Västra Götaland Sweden; ^3^ Centre for Inherited Metabolic Diseases, Karolinska University Hospital, Department of Medical Biochemistry and Biophysics Karolinska Institute Stockholm Sweden; ^4^ Neuropediatric Unit Astrid Lindgren Children's Hospital Stockholm Sweden; ^5^ Section for Pediatrics, Department of Clinical Sciences Lund University Lund Sweden

**Keywords:** adaptive behavior, epilepsy, intellectual disability, ketogenic diet, pyruvate dehydrogenase

## Abstract

**Objective:**

The aim of this study was to characterize intellectual and motor function, neurological features including epilepsy, treatment response, and adaptive behavior in patients with pyruvate dehydrogenase complex deficiency (PDCD) in Sweden.

**Methods:**

Forty‐two individuals with genetically confirmed PDCD (86% *PDHA1*‐related disease) were identified from a nationwide epidemiological study and were included in this cross‐sectional study comprising systematic neurological evaluations (*n* = 41) and caregiver interviews assessing adaptive behavior (*n* = 35).

**Results:**

Intellectual disability was detected in 33/42 (79%) individuals, while 31/35 (89%) demonstrated significant impairments in adaptive functioning. Although 27/39 (69%) were ambulatory, only 8/39 (21%) demonstrated age‐appropriate walking ability. Clinical signs of polyneuropathy were observed in 24/41 (59%), bulbar symptoms in 22/41 (54%), spasticity in 19/41 (46%), ataxia in 13/41 (32%), and dystonia in 9/41 (22%). Lifetime epilepsy was present in 16/41 (39%) of individuals. Ketogenic diet treatment, administered to 30 individuals, was effective in both prenatal‐ and postnatal‐onset disease. Seizure frequency decreased in individuals with epilepsy (8/9; 89%) and relapses of dystonia, ataxia, exercise intolerance, and lactic acidosis were prevented in all affected individuals (15/15). Improvements in communication and motor function were also noted.

**Discussion:**

Intellectual disability and deficits in adaptive behavior are frequent in PDCD, although cognitive outcomes are more heterogeneous among individuals with postnatal onset. Prenatal onset and epilepsy are associated with severe‐profound intellectual disability. Although most are ambulatory, motor deficits are frequent. A ketogenic diet treatment is a safe and effective therapeutic option, contributing to both seizure control and remission of neurological deterioration relapses.

## 1 Introduction

Pyruvate dehydrogenase complex is a mitochondrial matrix complex enzyme that connects glycolysis to the tricarboxylic acid cycle through catalyzing the conversion of pyruvate to acetyl coenzyme A [1, 2]. Pyruvate dehydrogenase complex deficiency (PDCD) is one of the most common causes of mitochondrial disease and lactic acidosis [3, 4]. The clinical spectrum includes: prenatal onset with brain lesions, growth restriction, microcephaly, and facial dysmorphism; neonatal presentations with hypotonia, encephalopathy, and lactic acidosis; and later, childhood or adult manifestations such as Leigh syndrome, episodic ataxia, dystonia, stroke‐like episodes, and polyneuropathy [5, 6, 7, 8]. Intellectual disability (ID), movement disorders, and seizures are reported in > 50% of all individuals [9]. The birth prevalence has been reported as 2.43 per 100,000 live births while the point prevalence is 0.44 per 100,000 [10].

ID is reported in 60%–83% of individuals with PDCD; however, prior studies have typically included small cohorts and lacked standardized assessments of cognitive and adaptive functioning [5, 6, 9]. Females are more likely to survive but tend to have poorer cognitive outcomes, typically ranging from severe to profound ID^5^. Motor manifestations have been primarily described in terms of spasticity and associated movement disorders [6, 11], whereas gross motor function and overall functional mobility remain poorly characterized. Epilepsy is a common early feature of PDCD, affecting 26%–57% of patients [5, 6, 11], yet its long‐term outcomes and prognostic factors have not been well studied.

Available treatment options for PDCD are limited and primarily focus on metabolic modulation. A ketogenic diet (KD) has been used to provide an alternative energy source by promoting ketone body utilization, thereby reducing dependence on pyruvate oxidation [12, 13, 14]. Given that thiamine pyrophosphate serves as an essential cofactor of the pyruvate dehydrogenase complex, thiamine supplementation is used to enhance residual enzymatic activity, though clinical benefit is only observed in a subset of patients [15, 16]. Nevertheless, current evidence regarding treatment efficacy and safety is largely based on individual case reports [13, 14, 16, 17, 18] and small patient series [12, 15].

The primary aim of our study was to determine the intellectual, epilepsy, and motor outcomes of patients with PDCD in Sweden. A secondary aim was to characterize adaptive‐behavior profiles within the cohort and assess treatment effects.

## 2 Methods

### 2.1 Study Population

**FIGURE 1:**
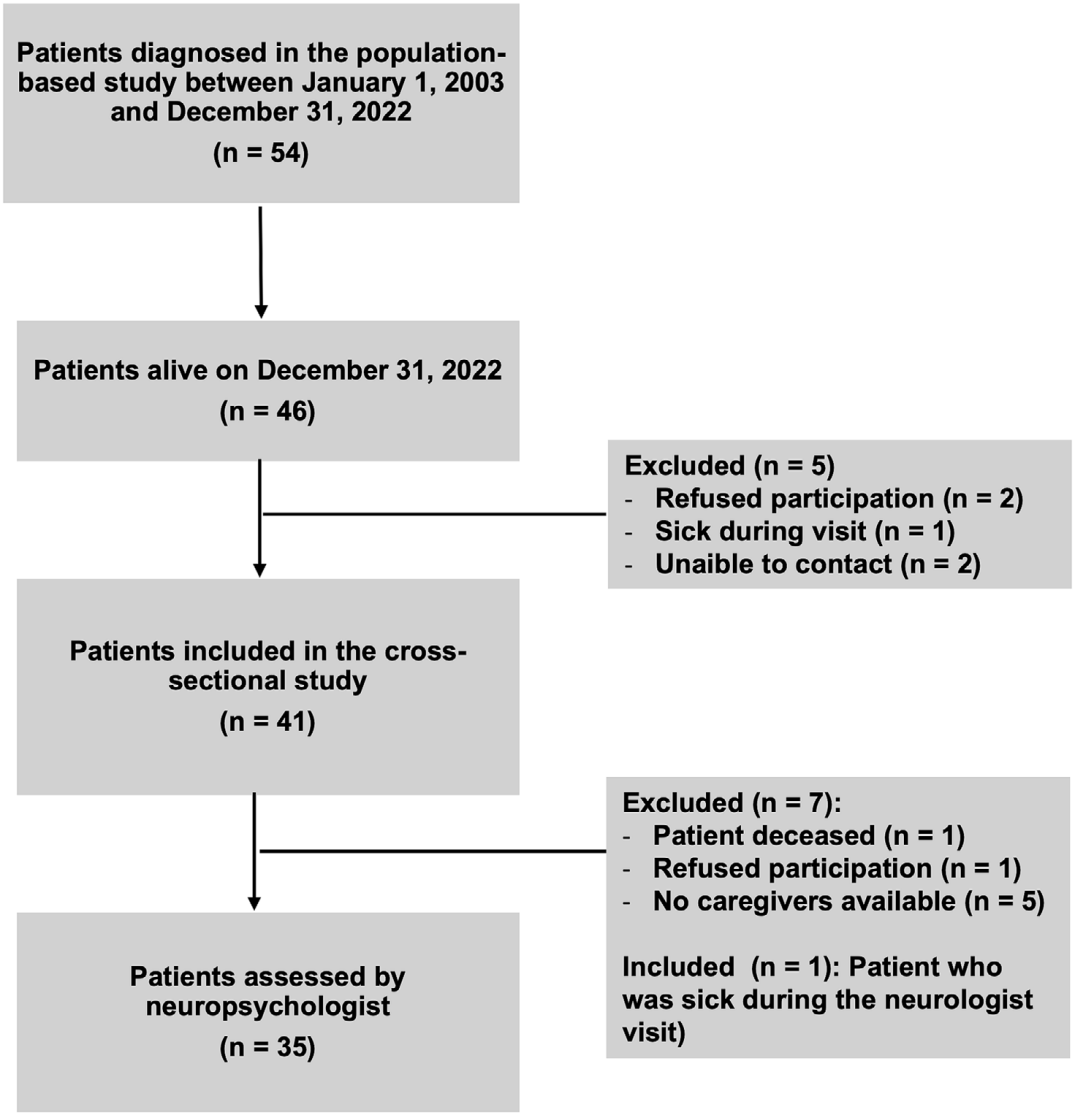
Flowchart for patient recruitment.

This cross‐sectional study was conducted between March and December 2022. Patients were recruited from a population‐based cohort including all individuals in Sweden diagnosed with PDCD between January 1, 2003 and December 31, 2022 (Figure 1) [10]. The study consisted of two parts: clinical evaluation, including a structured interview and examination by a pediatric neurologist (*n* = 41); and neuropsychological assessment (*n* = 35). Data on the presence and severity of intellectual disability (ID) were available for 42 patients: one individual was included in the neuropsychological assessment but not in the cross‐sectional clinical evaluation. Patients and/or their caregivers were contacted by their treating physicians.

### 2.2 Terminology

Prenatal‐onset disease was defined based on the presence of developmental or clastic lesions on neuroimaging indicative of prenatal onset [7], or by the presence of congenital microcephaly. Postnatal onset disease was defined as the appearance of clinical symptoms after birth, in the absence of radiological or clinical features suggestive of prenatal onset.

### 2.3 Data Collection and Assessment

Data regarding intellectual disability status and level were determined in 42 patients. A detailed developmental history was obtained and included a review of medical records, prior psychological assessments (if available), and documentation of any diagnoses of ID or global developmental delay. Neurological assessment also covered motor skills, speech and language development, cognition, personal and social development, and daily living skills as described by McDonald et al. [19].

Based on the available information, the presence and severity of ID were determined by consensus between a pediatric neurologist (A.S.) and a neuropsychologist (C.R.). Individuals were classified as having ID if there was evidence of significant deficits in intellectual functioning (i.e., intelligence quotient/developmental quotient < 70) and adaptive behavior consistent with DSM‐5 criteria [20].

Adaptive behavior was assessed in 35 patients. The caregivers of these patients with PDCD were interviewed with Vineland Adaptive Behavior Scales, Second Edition (VABS‐II) [21]. VABS‐II assesses adaptive behavior in four domains: communication, daily living skills, socialization, and motor (only investigated for children < 7 years of age). A total Adaptive Behavior Composite (ABC) score (mean 100, SD 15), reflecting overall functioning, was also calculated [21].

Neurological assessment was undertaken in 41 patients. A detailed neurological history and examination were obtained for each participant using a study‐specific case report form. Neurological examination included assessment of motor system (upper and lower limbs, and tendon reflexes), sensory system (superficial and deep sensation), coordination, movement disorders (ataxia and dystonia), and bulbar function (swallowing). The history included information on epilepsy and treatment. Seizure semiology and electroencephalogram findings were used to classify epileptic seizures according to International League Against Epilepsy criteria [22]. ‘Active’ epilepsy was defined as having had at least one epileptic seizure in the year before assessment. ‘Drug‐resistant epilepsy’ was defined as failure to achieve sustained seizure freedom after adequate trials of at least two appropriately chosen and tolerated antiseizure regimens, whether as monotherapy or in combination [23].

Gross motor function was classified using the 7‐level Gross Motor Function Classification for Metachromatic Leukodystrophy (GMFC‐MLD) where Level 0 is age‐appropriate walking ability and Level 6 is absence of any voluntary movement, including loss of head and trunk stability [24]. GMFC‐MLD was chosen instead of the Gross Motor Function Classification System as it also classifies gross motor function in individuals with normal gait. Ambulation is considered to be present when the individual is able to take at least five independent steps [25]. Clinical signs of polyneuropathy included pes cavus and at least one of the following clinical signs: muscle wasting, loss of vibration sense, diminished tendon reflexes, or sensory ataxia. The term “polyneuropathy” is used only if it has been confirmed by electroneurography.

All patients treated with a ketogenic diet (KD) were evaluated according to a national structured follow‐up protocol at treatment initiation, after 3 and 6 months, and every 6 months thereafter at one of the four University Hospitals in Sweden providing KD treatment. Data on treatment and efficacy was retrospectively extracted from medical records based on routine follow‐up evaluations conducted by the treating physicians. Treatment effectiveness was measured through changes in seizure frequency, motor function, communication (e.g., use of words, sounds, or eye contact) and relapsing episodes (intermittent movement disorders such as dystonia, ataxia, muscle weakness, or exercise intolerance) and was categorized on a 5‐point Likert scale from ‘very much improved’ to ‘very much worse’, as assessed by the treating physician. Reduction in seizure frequency was defined as a lower seizure frequency compared to baseline. Treatment safety was evaluated via reported side effects and biochemical investigations performed in a fasting state during routine follow‐up. Treatment compliance was assessed from ketosis based on 3‐hydroxybutyric acid (ketone bodies) levels measured at home and at the laboratory at follow‐up visits and dietary log. The KD was considered ‘classic’ when the diet was based on fat to protein and carbohydrate ratio, while modified ketogenic diet (MKD) was considered when other types of KD were used (e.g., modified Atkins diet, low glycemic index treatment, and the medium chain triglyceride diet) [26].

### 2.4 Analyses

Descriptive statistics (mean, median, SD) were used to describe scores on VABS‐II, epilepsy onset, age at clinical assessment, and treatment. Logistic regression analyses were performed to identify factors associated with ‘severe‐profound ID’. The factors included were sex (male vs. female), patient age (median split), lifetime epilepsy (yes/no), and disease form (prenatal‐onset vs. postnatal‐onset). All factors were first tested in univariable analysis, and factors associated at the *p* < 0.200 level were included in multivariable modeling [27]. Analyses were performed with IBM SPSS Version 29.0 (IBM Corp., Armonk, NY).

### 2.5 Ethics

The study was approved by the Ethical Review Board at Gothenburg University (#289–17, 106–04) and the Swedish Ethical Review Authority (Dnr 2019–05101, Dnr 2020–06408, and Dnr 2023–03887‐02). Caregivers provided written informed consent.

## 3 Results

Patient characteristics are shown in Table 1 with additional information provided in Table S1. Among the 42 individuals (31 females), 23 had prenatal‐onset and 19 had postnatal‐onset. Among the 36 individuals with *PDHA1‐*related disease, 19 had prenatal onset (18 females) and 17 had postnatal onset (10 females). Two individuals were identified with *PDHX*‐related disease (one prenatal‐onset female and one postnatal‐onset male), two with *DLAT*‐related disease (both prenatal‐onset males), and one with a *PDHB‐*related disease (postnatal‐onset male). The median age at neurological assessment was 13.8 years (95% confidence interval [95% CI]: 11–21.9 years; range: 1 month–82 years).

**TABLE 1:** Characteristics of patients with PDCD in Sweden.

Characteristic	Total (*n* = 41)	Prenatal (*n* = 22)	Postnatal (*n* = 19)
Sex, no.			
Female	30	19	11
Male	11	3	8
Median age at latest examinations, years	13.8 (95% CI: 11–21.9) [range: 0.1–82]		
Genetic variant, no.			
*PDHA1*	36	19	17
*PDHX*	2	1	1
*DLAT*	2	2	0
*PDHB*	1	0	1
Neurological signs			
Bulbar signs	22	14	8
Spasticity	19	13	6
Dystonia	9	6	3
Ataxia	13	3	10
Clinical signs of polyneuropathy	24	8	16
Ambulation, no.	27/39	11/20	16/19
GMFC‐MLD level^a^, no.			
0	8	4	4
1	19	7	12
2	6	4	2
3	0	0	0
4	1	1	0
5	1	0	1
6	4	4	0
Intellectual disability level, no.			
None	9	1	8
Mild	9	3	6
Moderate	5	4	1
Severe/profound	19	14	5
Epilepsy			
Lifetime prevalence, no.	16	10	6
Median age at onset, months	6 (95% CI, 0.7–24) [range: birth–32 years]		

Abbreviations: 95% CI, 95% confidence interval; GMFC‐MLD, Gross Motor Function Classification for Metachromatic Leukodystrophy; PDCD, pyruvate dehydrogenase complex deficiency.

^a^ GMFC‐MLD levels: 0 = walks independently with age‐appropriate quality; 1 = walks independently but with reduced quality/instability; 2 = walks only with support, fewer than five independent steps; 3 = sits independently, locomotion limited to crawling/rolling; 4 = sits independently with no locomotion or cannot sit independently but can crawl/roll; 5 = cannot sit or locomote, head control preserved; and 6 = no locomotion and no head or trunk control.

### 3.1 Intellectual Disability Status and Level of Adaptive Behavior

**FIGURE 2:**
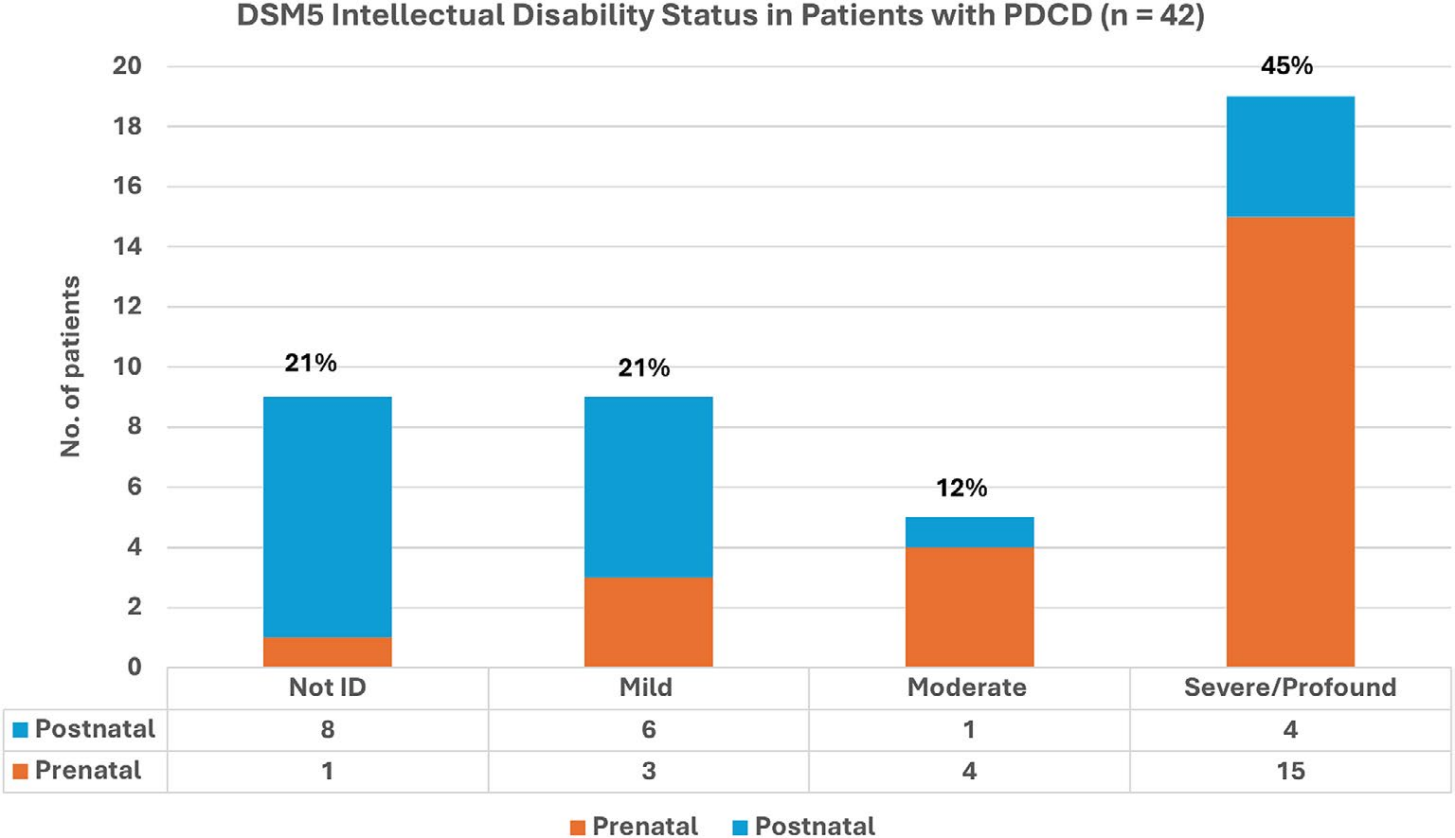
Intellectual disability status in patients with PDCD in Sweden. DSM5, Diagnostic and Statistical Manual of Mental Disorders 5; ID, intellectual disability; PDCD, pyruvate dehydrogenase complex deficiency; Prenatal, prenatal‐onset disease; postnatal, postnatal‐onset disease.

ID was present in 33 of 42 (79%) individuals, including 22 of 23 (96%) with prenatal‐onset and 11 of 19 (58%) with postnatal onset. Mild ID was present in nine of 42 (21%, prenatal onset = 3), moderate ID in five (12%, prenatal onset = 4), and severe or profound ID in 19 (45%, prenatal onset = 14) (Figure 2).

**FIGURE 3:**
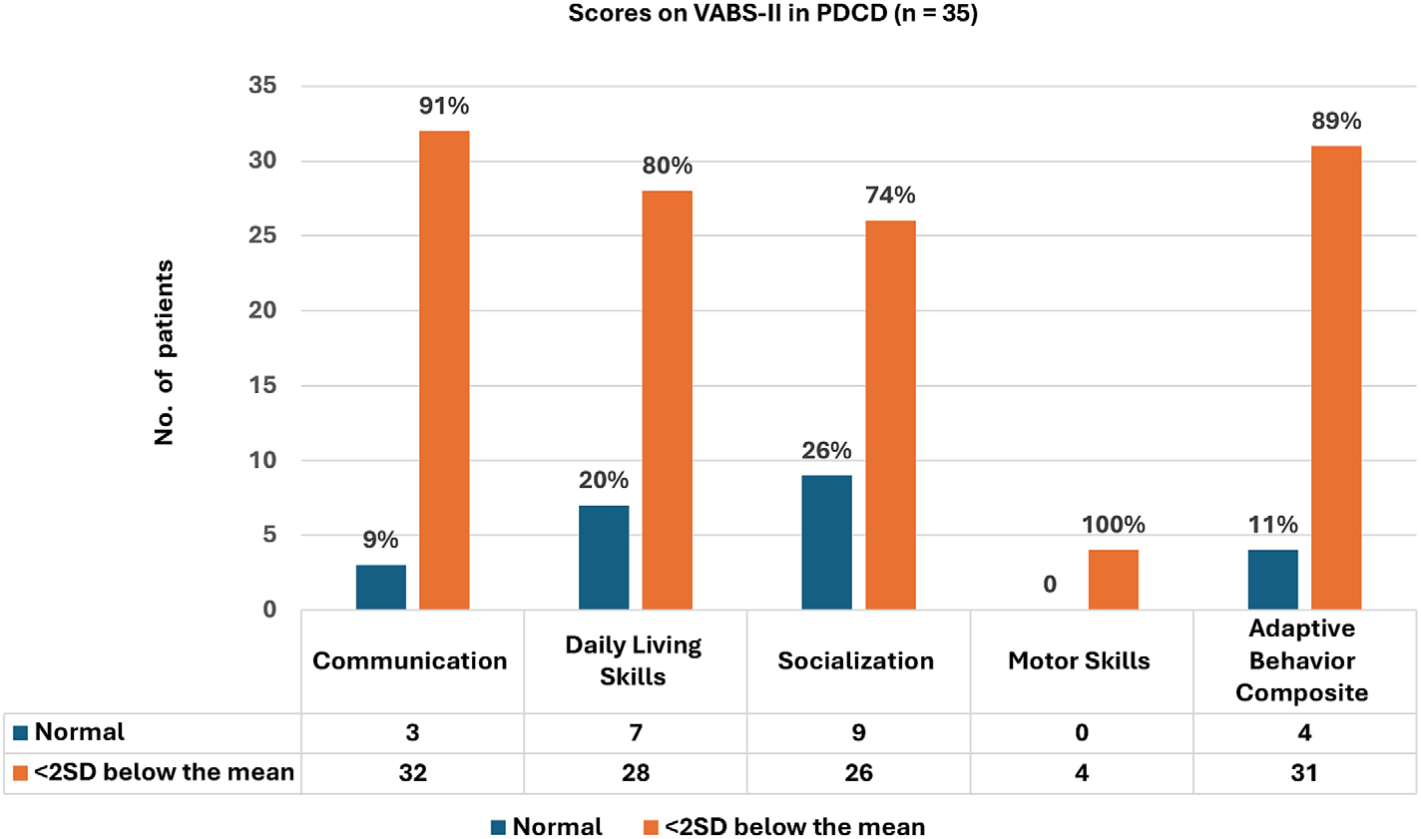
VABS‐II scores in patients with PDCD in Sweden. PDCD, pyruvate dehydrogenase complex deficiency; SD, standard deviation; VABS‐II, Vineland Adaptive Behavior Scales, Second Edition.

Mean scores on the VABS‐II are shown in Table 2 and Figure S1. ABC scores ranged from 20 to 87 (mean 40.54, SD 21.98). On VABS‐II, 11 (31%) individuals scored at the floor level, ie, the lowest possible score for ABC (Figure S2). Only four individuals did not have an ABC score less than two SDs below the mean, indicating that 31 of 35 (89%) individuals had significant difficulties (Figure 3). Socialization was the domain with the most individuals in the ‘normal’ range and the domain with highest mean score.

**TABLE 2:** Adaptive behavior scored on VABS‐II in patients with PDCD.

Behavior	No.	Range (min–max)	Mean (SD)
Communication	35	64 (20–84)	43.2 (19.82)
Daily living skills	35	75 (25–100)	49.1 (21.74)
Socialization	35	60 (25–85)	49.6 (19.51)
Motor skills	4	10 (42–52)	44.5 (5.00)
Adaptive behavior Composite	35	67 (20–87)	40.5 (21.98)

Abbreviations: max, maximum; min, minimum; PDCD, pyruvate dehydrogenase complex deficiency; SD, standard deviation; VABS‐II, Vineland Adaptive Behavior Scales, Second Edition (see reference by Reilly and colleagues (2023)).

The factors significantly associated with ‘severe‐profound ID’ on multivariable regression were the presence of prenatal disease onset (*p* = 0.011) and lifetime epilepsy (*p* = 0.004) (Table 3).

**TABLE 3:** Logistic regression analysis of factors associated with severe‐profound intellectual disability in patients with PDCD.

Factor	Univariable		Multivariable	
	OR (95% CI)	*p*	OR (95% CI)	*p*
Sex	0.183 (0.034–0.989)	0.049	0.252 (0.032–1.986)	0.190
Lifetime epilepsy	0.097 (0.023–0.414)	0.002	0.041 (0.005–0.369)	0.004
Prenatal vs. postnatal	0.142 (0.035–0.575)	0.006	0.056 (0.006–0.510)	0.011
Age median split	0.559 (0.164–1.911)	0.354	NA	NA

Abbreviations: 95% CI, 95% confidence interval; NA, not applicable; OR, odds ratio; PDCD, pyruvate dehydrogenase complex deficiency.

### 3.2 Motor Function and Neurological Findings

Ambulation was attained in 27 of 39 individuals (69%), while two were too young to have reached this developmental milestone. Eleven of 20 (55%) with prenatal‐onset PDCD were ambulatory, whereas 16 of 19 (84%) with postnatal‐onset PDCD were ambulatory.

Based on the GMFC‐MLD classification, individuals were rated as Grades 0 (*n* = 8), 1 (*n* = 19), 2 (*n* = 6), 3 (none), 4 (*n* = 1), 5 (*n* = 1), and 6 (*n* = 4). All grade 6 cases had prenatal onset (Table S1).

Bulbar symptoms were identified in 14 of 22 individuals (64%) with prenatal‐onset PDCD, of whom 12 underwent percutaneous endoscopic gastrostomy placement. Bulbar involvement was observed in eight of 19 individuals (42%) with postnatal‐onset PDCD, six of whom required percutaneous endoscopic gastrostomy insertion.

**FIGURE 4:**
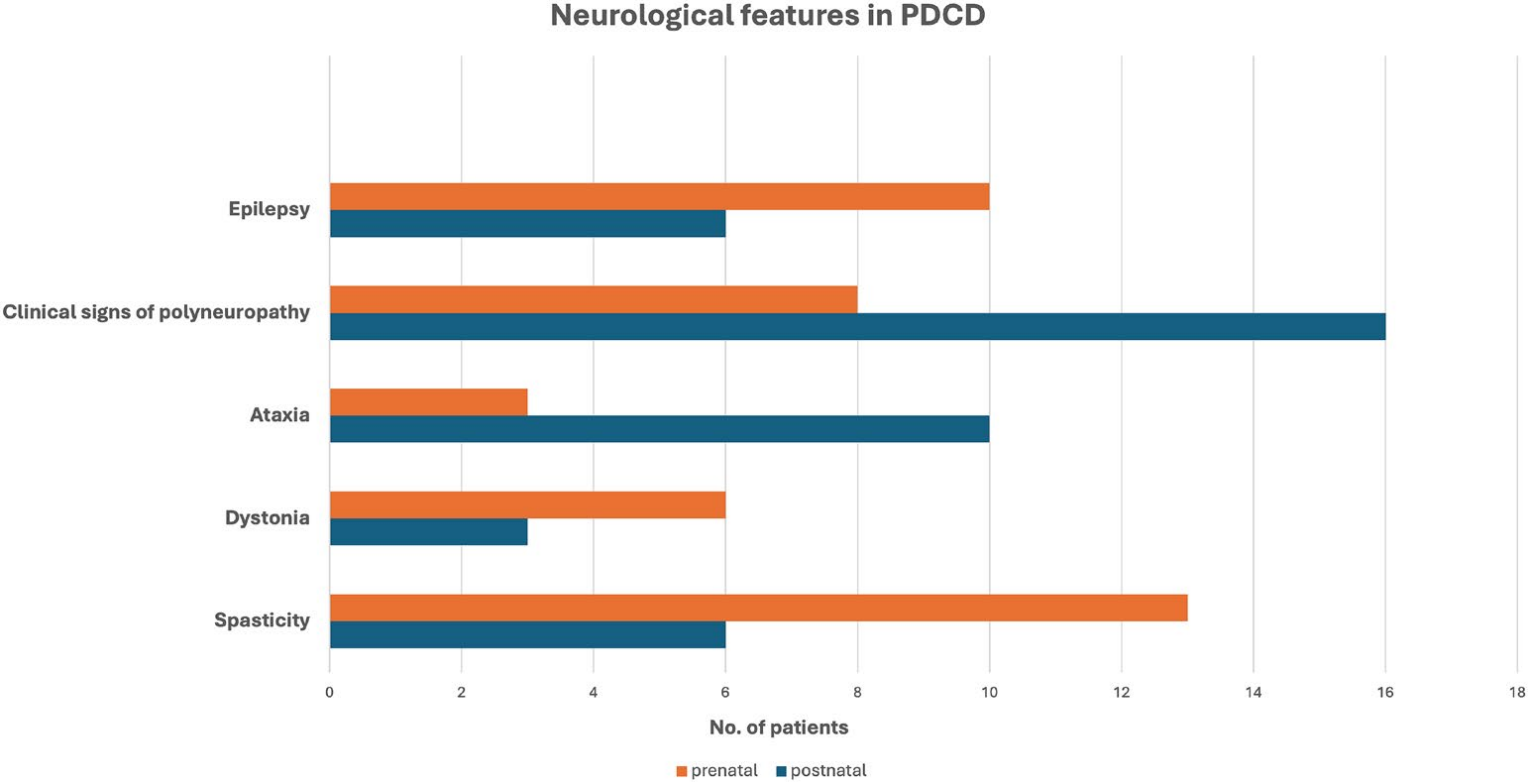
Neurological signs in patients with PDCD in Sweden. PDCD, pyruvate dehydrogenase complex deficiency. Prenatal, prenatal‐onset disease; Postnatal, postnatal‐onset disease.

Spasticity was observed in 19 individuals (46%, 13 prenatal‐onset), dystonia in nine (22%, 6 prenatal‐onset), ataxia in 13 (32%, 3 prenatal‐onset), and clinical signs of polyneuropathy in 24 (59%, 8 prenatal‐onset) (Figure 4). Four individuals underwent electroneurography, with findings consistent with a predominantly axonal sensorimotor neuropathy. Acquired microcephaly (< 2 SD for age) was seen in 20 of 41 individuals (49%) and short stature (< 2 SD for age) was documented in 15 of 41 (37%).

### 3.3 Epilepsy

A lifetime history of epilepsy was present in 16 of 41 individuals (39%) [10 prenatal‐onset]. Median age at first seizure was 6.5 months (95% CI, 2–24 months, range: birth to 32 years). At onset, 13 individuals had focal‐onset seizures (including focal‐to‐bilateral tonic–clonic in 7), two had generalized‐onset seizures (1 generalized tonic–clonic and 1 tonic), and one female presented with infantile epileptic spasms. Over time, mixed focal and generalized seizure types developed in three individuals (Table 1).

Drug‐resistant epilepsy developed in seven of ten individuals (70%) in the prenatal group and two of six patients (33%) in the postnatal group. Among patients with epilepsy, five of ten individuals (50%) with prenatal‐onset and five of six (83%) with postnatal‐onset were seizure‐free during the 12 months preceding latest assessment, one without anti‐seizure medication (Table S1).

### 3.4 Disease‐Specific Treatment

Thirty of 41 (73%) individuals had received KD treatment. The median duration of KD treatment among the individuals in the study was 7 years and 8 months (range, 1 month to 13.7 years).

Among the 22 individuals with prenatal‐onset, 18 (82%) were treated with KD: 14 received KD (ratio 2–4:1) and the remaining four were treated with MKD. Median age at start of treatment was 2 years and 8 months (interquartile range [IQR]: 1.3–7.6 years) and median blood ketone level was 2.4 mmol/L (IQR: 1.9–3.0 mmol/L). Six out of seven individuals with epilepsy and KD had a reduction in seizure frequency within 12 months of initiating treatment, four of them within 3 months, including two who achieved complete seizure cessation. At last follow‐up, four individuals with prenatal‐onset remained seizure‐free on KD monotherapy. Following treatment initiation, all individuals with intermittent dystonia (*DLAT* [*n* = 2] and *PDHA1* [*n* = 1]) and all individuals with exercise intolerance (*PDHA1* [*n* = 3]) remained relapse‐free. Improvements in motor function were observed in 15 individuals, 13 of which occurred within 3 months of treatment initiation. Improvements in communication were noted in 14 individuals, including 11 that occurred within 3 months.

Twelve of 19 (63%) individuals with postnatal‐onset disease were treated with KD: ten received classic KD (ratio 1.9–3:1) and the remainder received MKD. Median age at treatment initiation was 3 years and 11 months (IQR: 1.3–6.1 years) with a median ketone level of 2.5 mmol/L (IQR: 1.5–3.3 mmol/L). Of the four individuals with KD and lifetime epilepsy, only two had active epilepsy at the time of KD initiation and both became seizure‐free within 3 months of starting KD. One patient developed epilepsy after starting KD, during a period with low compliance to the diet, while another had no seizures at KD initiation and was able to discontinue antiseizure medications after starting the diet. All four individuals were seizure‐free at last follow‐up on KD monotherapy. Improvement in motor function was observed in nine individuals, which occurred within 3 months of treatment initiation in seven individuals, and improvement in communication was noted in eight individuals, which occurred in seven individuals within 3 months of treatment initiation. Following KD initiation, individuals with intermittent ataxia, muscle weakness, or lactic acidosis during infection (*n* = 9) either remained relapse‐free (*n* = 7) or exhibited a reduced frequency of relapses (*n* = 2).

**FIGURE 5:**
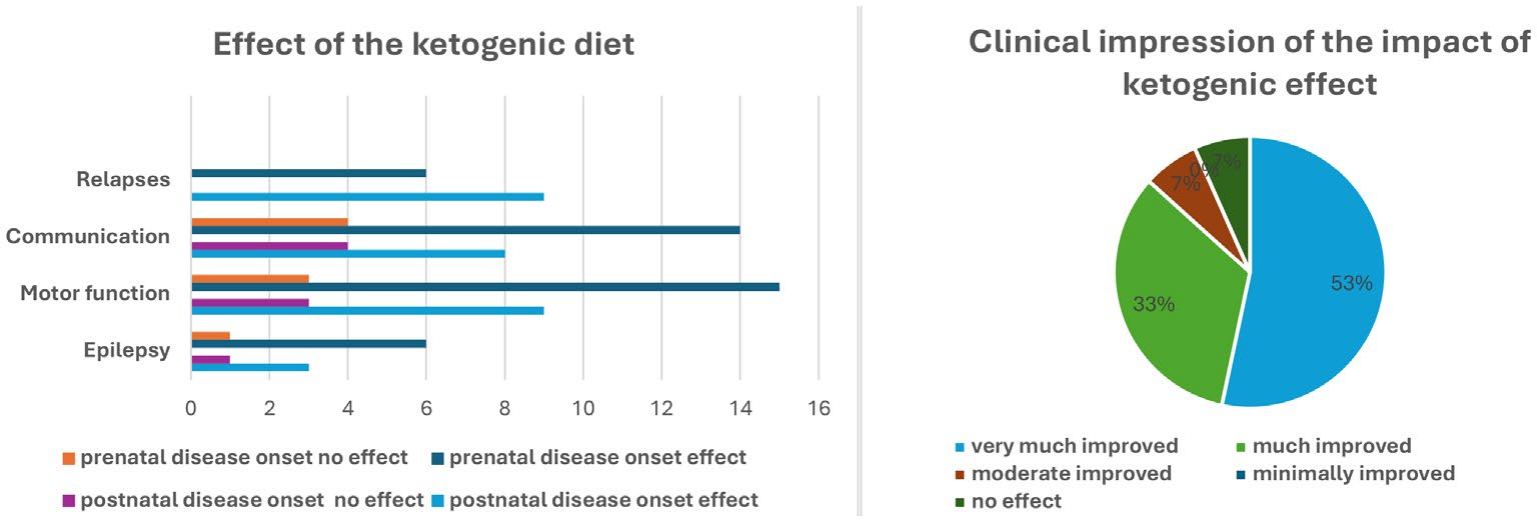
Effect of ketogenic diet in patients with PDCD in Sweden.

Of the 30 individuals treated with KD, 16 were rated as “very much improved”, ten as “much improved,” two as “moderately improved,” and two as “not improved” following KD (Figure 5). In total, 86% of individuals rated the overall effect of KD as “very much” or “much improved”.

The most common side effects of KD were constipation (*n* = 8) and nausea (*n* = 3). One individual developed pancreatitis and deep vein thrombosis and stopped treatment. Another individual, with poor compliance, discontinued treatment due to poor efficacy. One individual experienced transient metabolic acidosis at treatment initiation but was able to continue KD successfully.

Twenty‐eight individuals with *PDHA1*‐related disease received thiamine treatment (300–500 mg/day). Seven individuals reported a treatment effect, but only five received thiamine independently of KD. Among these, one female with prenatal‐onset disease reported a reduction in dystonia severity, while four individuals with postnatal‐onset disease reported decreased severity and duration of ataxia (Table S2).

## 4 Discussion

In the present study, ID was identified in 79% of individuals with PDCD, which is consistent with previous cohort studies [5, 6], but differed from the 57% found in a multicenter review study [11]. These discrepancies may be explained by differences in study design, as our population‐based, cross‐sectional study included a broader spectrum of disease severity, including individuals with milder phenotypes, whereas review studies rely on retrospectively reported cases from the literature, which are more likely to represent individuals with more severe phenotypes or include patients for whom ID status is incompletely reported.

Notably, the severity of ID varied depending on the timing of disease onset. Severe to profound ID was most prevalent among individuals with prenatal‐onset disease, whereas a substantial proportion of those with postnatal‐onset disease exhibited normal intellectual functioning. Preserved cognition in PDCD has previously been reported mainly in isolated case reports of individuals with intermittent movement disorders [18, 28, 29]. Our findings are consistent with these observations and further suggest that postnatal onset may be associated with a more favorable cognitive outcome. Along these lines, both prenatal‐onset disease and a history of epilepsy were associated with a more pronounced intellectual impairment, underscoring the importance of early disruption of neuronal energy metabolism as a key contributor to cognitive outcome in PDCD.

Assessment of adaptive functioning revealed a characteristic profile in which socialization emerged as a relative strength. A ‘floor effect’ was seen on the measure of adaptive behavior, with 31% of assessed individuals scoring at the lowest level on the Vineland composite score. This indicates that Vineland scoring may lack sensitivity for detecting differences among individuals with severe impairments in adaptive functioning. When a substantial proportion of participants cluster at the bottom of the scoring range, the instrument's ability to capture subtle variations or longitudinal changes becomes limited [30, 31]. Consequently, the Vineland ABC may be suboptimal for monitoring change or assessing treatment efficacy in this population, highlighting the need for more suitable instruments or alternative scoring methods, such as age equivalents and raw or growth score values or equivalents.

In comparison to a recent study [9], a higher proportion of individuals in our cohort were ambulatory, but only a small proportion demonstrated age‐appropriate walking ability, highlighting the significant impact of the disease on motor function. Spasticity and movement disorders were among the most frequent clinical features in our study, each observed in approximately half of the individuals, in line with previous reports [5, 6, 9]. Although congenital microcephaly was relatively uncommon, occurring in fewer than one‐fifth of individuals [9, 10], acquired microcephaly was observed in approximately half of the cohort. Clinical signs of polyneuropathy were present in nearly two‐thirds of individuals, a higher prevalence than previously reported [6, 9]. In our study, electrophysiological data were too limited. Nevertheless, our findings, together with previously published evidence, indicate that the neuropathy is likely to be predominantly axonal and sensorimotor [6, 32].

The prevalence of epilepsy in our cohort was 39%, consistent with previous reports [5, 6, 9]. Although generalized seizure types have been reported as more common in earlier PDCD cohorts, focal‐onset epilepsy was the predominant seizure phenotype at onset in our population [5, 6]. In contrast to a previous Japanese study reporting infantile epileptic spasms in approximately one‐third of females, we identified infantile spasms in only one female in our cohort [33]. However, in our population‐based cohort [10], which included deceased individuals, a total of five of 54 patients had infantile spasms, of whom three were females with *PDHA1* (3/30, 10%). Drug‐resistant epilepsy was significantly more common in individuals with prenatal‐onset than in those with postnatal‐onset, possibly reflecting distinct seizure mechanisms related to brain malformations in the former group and cerebral energy deficiency in the latter, as previously discussed [6, 17].

We observed a therapeutic effect of KD treatment in individuals with both prenatal‐ and postnatal‐onset PDCD, including marked reductions in seizure frequency as well as in relapses of ataxia, dystonia, lactic acidosis, and exercise intolerance. Improvements in communication and motor function were also noted. Although the antiseizure efficacy of KD in PDCD is relatively well established [12], data on its effects on motor symptoms remain limited [12, 14, 34], and the benefit of KD in prenatal‐onset disease has been questioned due to the presence of structural brain lesions [6, 35].

Thiamine responsiveness has been described in *PDHA1*‐related PDCD [15, 16, 17, 18]. Reported responders have predominantly exhibited postnatal‐onset phenotypes, including Leigh syndrome, relapsing ataxia, or dystonia, and have most often carried *PDHA1* variants affecting the thiamine pyrophosphate‐binding site [15, 16, 34, 36]. In the present study, none of the individuals who reported a clinical response to thiamine carried variants affecting the thiamine pyrophosphate‐binding site and none of the identified variants have previously been described as thiamine‐responsive. As in vitro functional studies were not performed and thiamine was administered concomitantly with the ketogenic diet, the observed treatment effects remain hypothetical. Nevertheless, studies have demonstrated that some thiamine‐responsive variants do not directly involve the cofactor‐binding site [15, 16, 37], suggesting that thiamine treatment could be considered in all individuals with *PDHA1* variants.

The strength of this study lies in its population‐based cross‐sectional design and the use a national structured follow‐up protocol for evaluating ketogenic diet treatment. An additional strength is the use of validated and widely used instruments, enabling the comparability between studies, although such instruments have limitations, as previously discussed regarding the Vineland scales. Another limitation is that the sample size was small, and data on treatment efficacy were collected retrospectively. In addition, data on adaptive behavior were unavailable for seven individuals and participants did not undergo standardized cognitive or developmental assessments.

In conclusion, this population‐based study systematically assessed the prevalence of ID, epilepsy, motor function, treatment effects, and adaptive behavior in PDCD. Most individuals exhibited significant impairments in intellectual function and adaptive behavior; however, nearly half of those with postnatal onset demonstrated normal cognition. Epilepsy was a common manifestation, and KD was shown to be a safe and effective therapy, associated with seizure reduction, prevention of disease relapses, and improvements in motor function and communication.

## Author Contributions

A.S. and N.D. contributed to conceptualization of the study and funding acquisition. A.S. and N.D. developed the methodology. A.S., C.R., and N.D. conducted the investigation. C.R. and N.D. supervised the study. A.S., C.R., and N.D. visualized the data. N.D. validated the study. A.S. curated the data and wrote the original draft of this manuscript. A.S. and C.R. conducted formal analysis and managed software. All authors were involved with reviewing and editing the manuscript.

## Funding

The study was supported by the Frimurare‐Barnhusdirektion Foundation (A.S.), Torbjörn Jebners Foundation (A.S.), Crown Princess Lovisa Foundation (A.S.), Linnea and Josef Carlssons Research Foundation (N.D., A.S.), and the Swedish state under the agreement between the Swedish government and the country councils (N.D.: ALFGBG‐1005213, A.S.: ALFGBG‐70150).

## Conflicts of Interest

The authors declare no conflicts of interest.

## Supporting information


**Table S1:** Characteristics of Patients with PDCD in Sweden.
**Table S2:** Impact of Ketogenic Diet and Thiamine Treatment in Patients with PDCD in Sweden.
**Figure S1:** Mean scores on Vineland Adaptive Behavior Scales, Second Edition (VABS‐II) total adaptive composite and individual domain scores for patients with PDCD in Sweden.
**Figure S2:** Vineland Adaptive Behavior Scales, Second Edition (VABS‐II) total adaptive composite (ABC) score vs. and for patients with PDCD in Sweden.

## Data Availability

The data that support the findings of this study are available from the corresponding author upon reasonable request.
