# Supplementary material for: Neurological, Neurodevelopmental and Treatment Outcomes in Patients With Pyruvate Dehydrogenase Complex Deficiency
Source: Ann Clin Transl Neurol. 2026 Aug 6:10.1002/acn3.70510. Online ahead of print. doi: 10.1002/acn3.70510 (PMC13445312; doi:10.1002/acn3.70510)
Supplement: Supplementary file 1 [file ACN3-9999-0-s001.docx]

**SUPPLEMENTAL MATERIAL**

Neurological and Neurodevelopmental Outcomes in Patients with Pyruvate Dehydrogenase Complex Deficiency by Antri Savvidou *et al*.

**Supplemental Table S1. Characteristics of Patients with PDCD in Sweden.**

prenatal

| Prenatal disease onset |  | | | | | **Upper motor neuron-movement disorders** | | | | **Lower motor neuron** |  | | **Epilepsy and antiepileptic treatment** | | | | |
| --- | --- | --- | --- | --- | --- | --- | --- | --- | --- | --- | --- | --- | --- | --- | --- | --- | --- |
|  | **Genetic variant** | **Gender** | **ID** | **Variant** | **Age at last examination** | **Bulbar signs^a^** | **Spasticity** | **Dystonia** | **Ataxia** | **ENG verified or clinical signs of polyneuropathy** | **Ambulation/**  **GMFC-MLD^b^** | **Intellectual disability level** | **Age at start** | **Semiology^c^** | **No. of ASMs used^d^** | **Active epilepsy** | **Active ASM treatment** |
|  | *PDHA1* | F | 1 | c.416delC | 14y3m | + | + | – | – | – | –/6 | Severe/profound | 2y | FBTC | 5 | + | CLOB, LAC |
|  |  | F | 2 | c.853_865dup13 | 1m | + | – | + | – | – | Too young | Severe/profound | – | – | NA | NA | NA |
|  |  | F | 3 | c.1142_1145dup | 11y | + | – | – | + | + | +/1 | Severe/profound | – | – | NA | NA | NA |
|  |  | F | 4 | c.1142_1145dup | 13y | + | – | – | + | + | +/1 | Severe/profound | 15m | FPC | 0 | – | KD |
|  |  | F | 5 | c.858_861dupTTAC | 10y | + | + | – | – | + | –/2 | Severe/profound | 7m | FIC | 0 | – | KD |
|  |  | F | 6 | c.904C>T | 11y | + | + | – | – | + | –/6 | Severe/profound | 6m | GES | 3 | – | KD |
|  |  | F | 7 | c.904C>T | 23y | + | + | + | – | – | –/2 | Severe /profound | 1w | FPC | 3 | – | KD |
|  |  | F | 8 | c.904C>T | 9y9m | + | + | – | – | + | –/2 | Severe/profound | 3m | FPC | 2 | + | LAM, KD |
|  |  | F | 9 | c.904C>T | 9y | + | + | + | – | – | –/6 | Severe/profound | 6m | FPC | 3 | + | VAL, TOP, KD |
|  |  | F | 10 | c.871G>A | 28y | + | + | – | – | + | –/4 | Severe/profound | 3d | FBTC | 1 | – | – |
|  |  | F | 11 | c.493G>A | 21y11m | – | + | – | – | – | +/1 | Severe/profound | – | – | NA | NA | NA |
|  |  | F | 12 | c.355C>T | 10m | + | – | – | – | – | Too young | Moderate | – | – | NA | NA | NA |
|  |  | F | 13 | c.1040_1048dup9 | 23y9m | + | + | + | – | – | –/6 | Severe/profound | 0d | GTC | 4 | + | LAM, OXC |
|  |  | F | 14 | c.863G>A | 13y2m | – | + | + | – | – | +/1 | Severe/profound | – | – | NA | NA | NA |
|  |  | F | 15 | c.380G>A | 27y7m | + | + | + | – | – | –/2 | Moderate | 3w | FBTC | 5 | + | CARB, LAM |
|  |  | F | 16 | c.455C>T | 12y8m | – | – | – | – | + | +/0 | Mild | – | – | NA | NA | NA |
|  |  | F | 17 | c.430G>A | 8y1m | – | – | – | + | – | +/1 | Mild | – | – | NA | NA | NA |
|  |  | F | 18 | c.728A>G | 30y4m | – | – | – | – | + | +/0 | Mild | – | – | NA | NA | NA |
|  |  | M | 19 | c.447A>G | 6y8m | – | + | – | – | – | +/1 | None | – | – | NA | NA | NA |
|  | *PDHX* | F | 20 | c.1336C>T | 29y5m | + | + | – | – | – | +/1 | Severe/profound | – | – | NA | NA | NA |
|  | *DLAT* | M | 21 | c.470T>G | 11y11m | – | – | – | – | – | +/0 | Moderate | – | – | NA | NA | NA |
|  |  | M | 22 | c.470T>G | 9y4m | – | – | – | – | – | +/0 | Moderate | – | – | NA | NA | NA |
|  | *PDHA1*  Postnatal disease onset | F | 23 | c.1133G>A | 25y | + | – | + | – | + | –/5 | Severe/profound | 5y | FBTC | 1 | + | LAM |
|  |  | F | 24 | c.1132C>T | 5y6m | + | + | – | + | – | +/1 | Moderate | – | – | NA | NA | NA |
|  |  | F | 25 | c.380G>A | 9y8m | + | + | – | + | + | –/2 | Severe/profound | – | – | NA | NA | NA |
|  |  | F | 26 | c.380G>A | 14y8m | + | + | + | + | + | +/1 | Mild | – | – | NA | NA | NA |
|  |  | F | 27 | c.410A>G | 20y | + | + | – | – | + | +/1 | Mild | – | – | NA | NA | NA |
|  |  | F | 28 | c.615C>G | 4y9m | – | – | – | – | + | +/1 | Mild | – | – | NA | NA | NA |
|  |  | F | 29 | c.407C>T | 52y | – | – | – | + | + | +/1 | None | – | – | NA | NA | NA |
|  |  | F | 30 | c.410A>G | 54y | – | – | – | – | + | +/1 | None | 8y | FBTC | 3 | – | LAM |
|  |  | F | 31 | c.262C>T | 45y | – | – | – | – | + | +/0 | None | – | – | NA | NA | NA |
|  |  | F | 32 | c.410A>G | 82y | – | – | – | – | + | +/1 | None | – | – | NA | NA | NA |
|  |  | M | 33 | c.1132C>T | 13y | + | – | + | + | + | –/2 | Severe/profound | 2m | GT | 0 | – | KD |
|  |  | M | 34 | c.407C>T | 22y | + | – | – | + | + | +/1 | Mild | – | – | NA | NA | NA |
|  |  | M | 35 | c.1100A>G | 30y | – | + | – | + | + | +/1 | Mild | 32y | FPC | 1 | – | KD |
|  |  | M | 36 | c.407C>T | 20y | – | – | – | + | + | +/1 | Mild | – | – | NA | NA | NA |
|  |  | M | 37 | c.447A>T | 17y | – | – | – | – | + | +/1 | None | – | – | NA | NA | NA |
|  |  | M | 38 | c.262C>T | 7y | – | – | – | – | – | +/0 | None | 3y | FBTC | 1 | – | KD |
|  |  | M | 39 | c.570C>T | 13y9m | – | – | – | – | – | +/0 | None | – | – | NA | NA | NA |
|  | *PDHX* | M | 40 | c.1336C>T | 15y | + | + | – | + | + | +/1 | Severe/profound | 7m | FIC | 2 | – | KD |
|  | *PDHB* | F | 41 | c.301A>G, c.790G>A | 5y | – | – | – | + | + | +/0 | None | – | – | NA | NA | NA |

^a^Drooling, dysarthria, or dysphagia.

^b^GMFC-MLD levels: 0 = walks independently with age-appropriate quality; 1 = walks independently but with reduced quality/instability; 2 = walks only with support, fewer than five independent steps; 3 = sits independently, locomotion limited to crawling/rolling; 4 = sits independently with no locomotion or cannot sit independently but can crawl/roll; 5 = cannot sit or locomote, head control preserved; and 6 = no locomotion and no head or trunk control.

^c^Semiology defined according to International League Against Epilepsy.

^d^KD is not included in the number of ASMs used.

Abbreviations: +, yes; –, no; ASM, anti-seizure medication; CARB, carbamazepine; CLOB, clobazepam; d, day; E, electroneurogram; F, female; FBTC, focal to bilateral tonic clonic seizure; FIC, focal with impaired consciousness seizure; FPC, focal preserved consciousness seizure; GES, generalised epileptic spasms; GT, generalized tonic seizure; GTC, generalized tonic-clonic; ID, identification number; KD, ketogenic diet; LAC, lacosamide; LAM, lamotrigine; M, male; m, month; NA, not applicable; OXC, oxcarbazepine; PDCD, pyruvate dehydrogenase complex deficiency; TOP, topiramate; VAL, sodium valproate; y, year.

**Supplemental Table S2. Impact of Ketogenic Diet and Thiamine Treatment in Patients with PDCD in Sweden.**

|  |  | | | | **Ketogenic diet** | | | | | | | **Thiamine** |
| --- | --- | --- | --- | --- | --- | --- | --- | --- | --- | --- | --- | --- |
| Prenatal disease onset | **Genetic variant** | **Gender** | **ID** | **Age last examination** | **KD type** | **Age at KD start** | **Epilepsy/ time effect** | **Motor function/time effect** | **Communication/**  **time effect** | **Relapses/time effect/symptom improved** | **Clinical impression of Treatment effectiveness** | **Treatment/**  **effect** |
|  | *PDHA1* | F | 1 | 14y3m | classic | 4y2m: stopped after 1y | +/3m | +/3m | +/3m | –/Infections | Much improved | +/– |
|  |  | F | 2 | 1m | classic | 0y0m | NA | +/1m | +/1m | NA | Very much improved | +/NA |
|  |  | F | 3 | 11y | classic | 7y7m | NA | +/3m | +/3m | NA | Very much improved | +/– |
|  |  | F | 4 | 13y | classic | 1y7m | +/3m | +/6m | +/6–9m | NA | Much improved | +/– |
|  |  | F | 5 | 10y | classic | 0y7m | +/3m | +/3m | +/3m | NA | Very much improved | NA/NA |
|  |  | F | 6 | 11y | classic | 1y4m | +/3m | +/3m | +/12m | +/9m/infections | Much improved | +/– |
|  |  | F | 7 | 23y | classic | 15y3m | +/12m | +/3m | +/3m | +/3m/exercise intolerance | Very much improved | NA/NA |
|  |  | F | 8 | 9y9m | classic | 0y1m | +/6m | +/12m | +/>12m | NA | Much improved | +/– |
|  |  | F | 9 | 9y | classic | 0y0.5m | – | – | – | NA | No effect | +/+ |
|  |  | F | 10 | 28y | NA | NA | NA | NA | NA | NA | NA | +/– |
|  |  | F | 11 | 21y11m | classic | 14y5m | NA | +/3m | +/3m | +/3m/exercise intolerance | Very much improved | +/– |
|  |  | F | 12 | 10m | classic | 0y8m | NA | +/3m | +/3m | NA | Very much improved | – |
|  |  | F | 13 | 23y9m | NA | NA | NA | NA | NA | NA | NA | +/– |
|  |  | F | 14 | 13y2m | classic | 2y8m | NA | +/3m | +/3m | NA | Much improved | +/– |
|  |  | F | 15 | 27y7m | NA | NA | NA | NA | . | NA | NA | –/NA |
|  |  | F | 16 | 12y8m | classic | 2y | NA | +/3m | +/3m | +/3m/infection | Much improved | +/– |
|  |  | F | 17 | 8y1m | classic | 1y9m | NA | +/3m | +/3m | +/3m/exercise intolerance | Very much improved | +/– |
|  |  | F | 18 | 30y4m | MKD | 19y | NA | +/3m | +/3m | +/3m/intermittent dystonia | Very much improved | +/+ |
|  |  | M | 19 | 6y8m | NA | NA | NA | NA | NA | NA | NA | +/– |
|  | *PDHX* | F | 20 | 29y5m | MKD | 12y6m: stopped after 2y | NA | – | – | NA | No effect | NA/NA |
|  | *DLAT* | M | 21 | 11y11m | MKD | 5y8m | NA | +/3m | +/ | +/3m/intermittent dystonia | Much improved | NA/NA |
|  |  | M | 22 | 9y4m | MKD | 3y1m | NA | – | – | +/<3m/intermittent dystonia | Much improved | NA/NA |
| Postnatal disease onset | *PDHA1* | F | 23 | 25y | NA | NA | NA | NA | NA | NA | . | +/– |
|  |  | F | 24 | 5y6m | classic | 0y8m | NA | +/3m | +/3m | +/3m/intermittent muscle weakness | Very much improved | +/NA |
|  |  | F | 25 | 9y8m | classic | 1y | NA | – | +/3m | NA | Moderate improved | +/NA |
|  |  | F | 26 | 14y8m | classic | 6y2m | NA | +/3m | +/3m | +/3m/infections | Very much improved | +/NA |
|  |  | F | 27 | 20y | classic | 8y | NA | +/3m | +/3m | +/3m/episodic ataxia | Much improved | +/– |
|  |  | F | 28 | 4y9m | classic | 4y | NA | NA | NA | +/3m/episodic ataxia | Very much improved | +/+ |
|  |  | F | 29 | 52y | NA | NA | NA | NA. | NA | NA | NA | –/NA |
|  |  | F | 30 | 54y | NA | NA | NA | NA | NA | NA | NA | –/NA |
|  |  | F | 31 | 45y | NA | NA | NA | NA | NA | NA | NA | –/NA |
|  |  | F | 32 | 82y | NA | NA | NA | NA | NA | NA | NA | –/NA |
|  |  | M | 33 | 13y | classic | 0y6.5m | +/3m | +/3m | +/3m | +/3m/lactic acidosis infections | Much improved | +/– |
|  |  | M | 34 | 22y | NA | NA | NA | NA | NA | NA | NA | +/+ |
|  |  | M | 35 | 30y | MKD | 24y5m | NA | +/3m | +/3m | +/3m/intermittent muscle weakness | Very much improved | +/+ |
|  |  | M | 36 | 20y | NA | NA | NA | NA | NA | NA | NA | +/+ |
|  |  | M | 37 | 17y | MKD | 3y5m | NA | +/ENG verified | NA | +/3m/intermittent ataxia | Very much improved | +/– |
|  |  | M | 38 | 7y | classic | 3y10m | +/3m | +/3m | +/3m | +/3m/intermittent ataxia | Very much improved | +/– |
|  |  | M | 39 | 13y9m | classic | 11y10m | NA | NA | NA | +/3m/intermittent ataxia | Very much improved | +/+ |
|  | *PDHX* | M | 40 | 15y | classic | 5y6m | NA | +/12m | +/12m | NA | Moderate improved | NA/NA |
|  | *PDHB* | F | 41 | 5y | classic | 1y3m | NA | +/1w | NA | +/1w intermittent muscle weakness | Very much improved | NA/NA |

Abbreviatons: +, yes; –, no; ENG, electroneurogram; F, females; KD, ketogenic diet; m, month; M, males; MKD, modified ketogenic diet; NA, not applicable/available; y, year.

**Supplemental Figure S1: Mean scores on Vineland Adaptive Behavior Scales, Second Edition (VABS-II) total adaptive composite and individual domain scores for patients with PDCD in Sweden.**

**Supplemental Figure S2: Vineland Adaptive Behavior Scales, Second Edition (VABS-II) total adaptive composite (ABC) score vs. and for patients with PDCD in Sweden.**
